# Intraosseous extradural meningioma of the frontal bone

**DOI:** 10.11604/pamj.2014.17.69.3844

**Published:** 2014-01-28

**Authors:** Ali Akhaddar, Hassan Ennouali

**Affiliations:** 1 Department of Neurosurgery, Avicenne Military Hospital, Marrakech, Morocco; 2 University of Mohammed V Souissi, Rabat, Morocco; 3 Department of Radiology, Mohammed V Military Teaching Hospital, Rabat, Morocco

**Keywords:** Meningioma, tumor, extradural, CT-scan

## Image in medicine

A 37-year-old woman, previously healthy with no history of trauma, presented with a scalp swelling of the left frontal region. The swelling was present for the last one year and had gradually increased in size with recent localized headache. The patient had no neurologic deficit. Local examination showed a painful local swelling of about 25 mm in diameter in the left frontal area and appeared to arise from the bone. Hematological and biochemical investigation results were within the normal range. Computed tomography scan revealed a left frontal intradiploic mass with bone defect (A). Magnetic resonance imaging showed that the lesion was soft with homogeneous enhancement following gadolinium injection. There was erosion of both inner and outer table of the cranial vault with extra cranial and intracranial extradural extension (B and C). The tumor was excised completely and the surrounding bone was removed (D), followed by cranial reconstruction. There was no intradural extension of the lesion. Histologically, the tumor was diagnosed as meningothelial meningioma. The patient has been well for 1 year following the operation with no evidence of recurrence. Meninigiomas are primary tumors of arachnoids cell layers and the lesions are in purely settled intradural locations. Meningiomas in extradural locations are very rare and should be considered in the differential diagnosis of osteolytic skull vault lesions.

**Figure 1:**
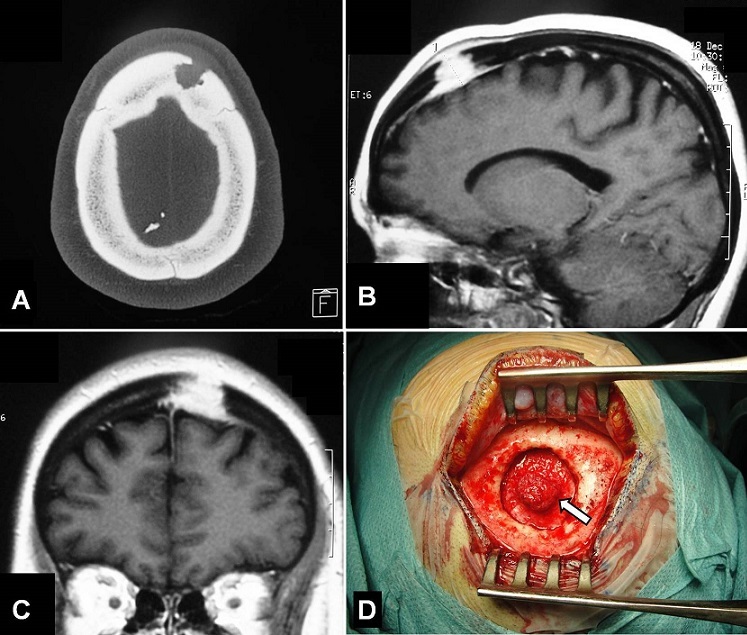
A) Axial CT-scan (bony window) revealing the left frontal bone defect; B and C) Sagittal and coronal T1-weighted MRI after gadolinium injection showing homogeneous enhancement of the lesion which was extradural and intraosseous with extra cranial and intracranial extension; D) Operative view of the tumor (arrow) after removing of the surrounding bone

